# Targeting Adrenomedullin in Oncology: A Feasible Strategy With Potential as Much More Than an Alternative Anti-Angiogenic Therapy

**DOI:** 10.3389/fonc.2020.589218

**Published:** 2021-01-06

**Authors:** Ramiro Vázquez, Maria E. Riveiro, Caroline Berenguer-Daizé, Anthony O’Kane, Julie Gormley, Olivier Touzelet, Keyvan Rezai, Mohamed Bekradda, L’Houcine Ouafik

**Affiliations:** ^1^ Preclinical Department, Early Drug Development Group (E2DG), Boulogne-Billancourt, France; ^2^ Center for Genomic Science of IIT@SEMM, Fondazione Istituto Italiano di Tecnologia (IIT), Milan, Italy; ^3^ Aix Marseille University, CNRS, INP, Institute of NeuroPhysiopathology, Marseille, France; ^4^ Discovery and Scientific Affairs Department, Fusion Antibodies plc., Belfast, United Kingdom; ^5^ Department of Radio-Pharmacology, Institute Curie-René Huguenin Hospital, Saint-Cloud, France; ^6^ APHM, CHU Nord, Service de Transfert d’Oncologie Biologique, Marseille, France

**Keywords:** adrenomedullin, angiogenesis, cancer, metastasis, AM_1_ and AM_2_, RAMP1-3

## Abstract

The development, maintenance and metastasis of solid tumors are highly dependent on the formation of blood and lymphatic vessels from pre-existing ones through a series of processes that are respectively known as angiogenesis and lymphangiogenesis. Both are mediated by specific growth-stimulating molecules, such as the vascular endothelial growth factor (VEGF) and adrenomedullin (AM), secreted by diverse cell types which involve not only the cancerogenic ones, but also those constituting the tumor stroma (i.e., macrophages, pericytes, fibroblasts, and endothelial cells). In this sense, anti-angiogenic therapy represents a clinically-validated strategy in oncology. Current therapeutic approaches are mainly based on VEGF-targeting agents, which, unfortunately, are usually limited by toxicity and/or tumor-acquired resistance. AM is a ubiquitous peptide hormone mainly secreted in the endothelium with an important involvement in blood vessel development and cardiovascular homeostasis. In this review, we will introduce the state-of-the-art in terms of AM physiology, while putting a special focus on its pro-tumorigenic role, and discuss its potential as a therapeutic target in oncology. A large amount of research has evidenced AM overexpression in a vast majority of solid tumors and a correlation between AM levels and disease stage, progression and/or vascular density has been observed. The analysis presented here indicates that the involvement of AM in the pathogenesis of cancer arises from: 1) direct promotion of cell proliferation and survival; 2) increased vascularization and the subsequent supply of nutrients and oxygen to the tumor; 3) and/or alteration of the cell phenotype into a more aggressive one. Furthermore, we have performed a deep scrutiny of the pathophysiological prominence of each of the AM receptors (AM_1_ and AM_2_) in different cancers, highlighting their differential locations and functions, as well as regulatory mechanisms. From the therapeutic point of view, we summarize here an exhaustive series of preclinical studies showing a reduction of tumor angiogenesis, metastasis and growth following treatment with AM-neutralizing antibodies, AM receptor antagonists, or AM receptor interference. Anti-AM therapy is a promising strategy to be explored in oncology, not only as an anti-angiogenic alternative in the context of acquired resistance to VEGF treatment, but also as a potential anti-metastatic approach.

## Introduction

The term cancer comprises different types of pathologies characterized by uncontrolled proliferation of cells that, with the exception of those of hematological or lymphatic origin, give place to malignant tumor masses. Primary tumors grow supported by new vascularization resulting from pre-existing capillaries in a sequence of events that are collectively known as angiogenesis. This process is triggered in response to spontaneous or induced tissue hypoxia, a common phenomenon in solid tumors. These new vessels are also used by cancer cells to spread to other sites within the body after acquiring invasive potential, thereby causing metastasis and, without intervention, death. Surgery and radiotherapy constitute the first approaches in the treatment of localized tumors while systemic agents (chemotherapy, hormone and biological therapies) are the choice to confront the metastatic setting.

In this context, the discovery of tumor angiogenesis opened a new path in fighting cancer. Hypoxia-inducible factor-1α (HIF-1α) is the master switch of the cell machinery required to face O_2_-lacking periods in physiological and pathological conditions. One of its target genes is that which encodes for the vascular endothelial growth factor (VEGF), the best characterized angiogenic promoter, involved in the modulation of vessel permeability and remodeling, and endothelial cell survival, proliferation and migration (1). The current angiogenesis-targeting approaches approved in clinical practice are: 1) VEGF-blocking monoclonal antibodies (bevacizumab/Avastin^®^); 2) decoy receptors, ‘VEGF-trap’ (aflibercept/Zaltrap^®^); 3) tyrosine kinase inhibitors (sunitinib/Sutent^®^, sorafenib/Nexavar^®^, axitinib/Inlyta^®^); and 4) monoclonal antibodies targeting VEGF receptors (ramucirumab/Cyramza^®^) (2). These agents are being used in the treatment of breast, colorectal, hepatocellular, gastric, and lung among other cancers (2), increasing the effectiveness of conventional chemotherapy. However, a significant number of preclinical and clinical observations have shown that the process of angiogenesis is far from being clearly understood. Furthermore, this approach is not effective in all cancers and often has only limited impact on patient’s overall survival, which, added to the occurrence of frequent drug toxicity and the development of resistance, support the necessity to explore novel strategies aiming to influence alternative factors involved in tumor angiogenesis. In this regard, additional approaches are being tested in preclinical and clinical trials including: angiopoietins (Ang), epidermal growth factor (EGF), fibroblast growth factors (FGF1 and FGF2), hepatocyte growth factor (HGF), platelet-derived growth factor C (PDGF-C), or agents targeting angiogenesis indirectly by inhibiting oncogenic pathways (e.g., HER2, PI3k/AkT/mTOR, and mutated EGF receptor) or hormone signaling (3).

Adrenomedullin –onwards AM– is a regulatory peptide whose involvement in tumor progression and metastasis has become more evident in recent years. The whole literature supports the idea of AM as a survival factor for tumor cells, which can be produced either by the malignant cells themselves or by those located in adjacent/surrounding stroma. In general, AM expression is upregulated by hypoxia, and the excessive production of this peptide is associated with poorer prognosis in cancer patients (4, 5). Moreover, recent reports indicate that AM could be a master regulator upstream of the VEGF pathway and even induce HIF-1α expression, therefore attracting much interest as a therapeutic strategy (6, 7).

Here, we review the physiological and pathological processes mediated by AM, analyzing the advantages that the employment of anti-AM therapy may offer in oncology.

## AM in Physiological Context

### AM, Calcitonin-Like Peptides, and Their Physiological Roles

**Figure 1:**
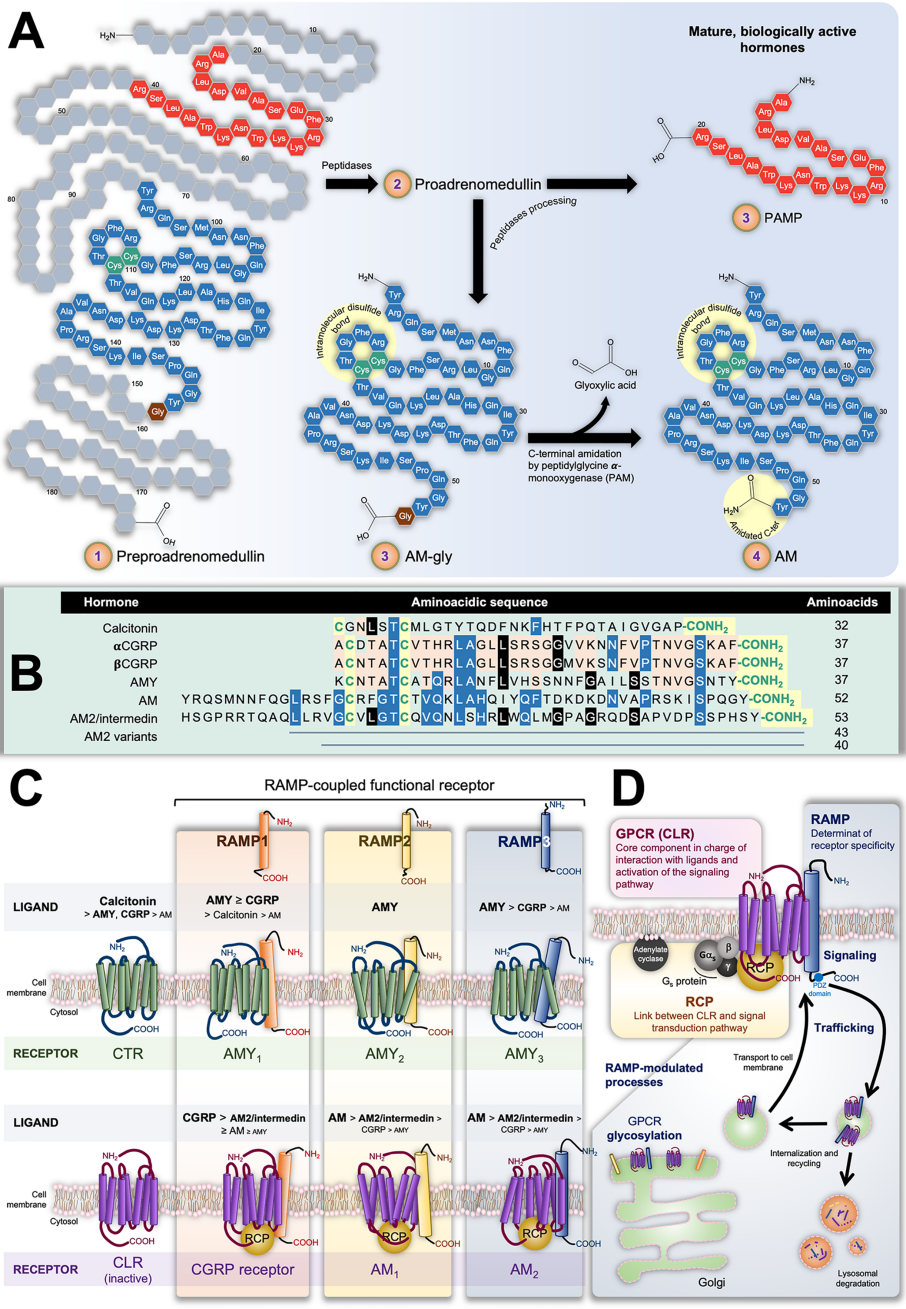
**(A)** Scheme representing preproadrenumedullin processing to give rise to the mature biologically active peptides adrenomedullin (AM) and proadrenomedullin N-terminal 20 peptide (PAMP), a potent angiogenic agent. Common structural features of the calcitonin-like peptide family (i.e., disulfide bond and amidated C-terminal) are highlighted by yellow circles. **(B)** Sequences of human components of this family. As in **(A)**, conserved characteristic structures are highlighted in yellow. Different colors indicate position-shared amino acids amongst the AM (blue), AM2/Intermedin variants (black), calcitonin gene-related peptide CGRP), and amylin (AMY) (orange) peptides. **(C)** Receptors with their decreasing affinity for different ligands. The interaction of calcitonin receptor (CTR) and calcitonin-receptor like receptor (CLR) with receptor-activity modifying proteins (RAMPs) have been very well studied in terms of the ability of the former group to alter the GPCR specificity by acting on their ligand affinity. The CTR binds calcitonin whilst presenting low affinity for AMY and CGRP. In contrast, it forms three AMY-high-affinity and calcitonin-low-affinity receptors (AMY_1_, AMY_2_, and AMY_3_) in the presence of RAMP1, RAMP2, or RAMP3, respectively. On the other hand, CLR itself is not able to bind any known ligand. Nonetheless, the CLR/RAMP1 dimer results in a high-affinity CGRP receptor, while its combination with RAMP2 or RAMP3 respectively produces the AM_1_ and AM_2_ subtypes of the AM receptor. Both CLR/RAMP1, AM_1_ and AM_2_ also show moderate affinity for AM2/intermedin. **(D)** Constituents of the AM_2_ and their role in the tripartite complex.

The isolation and characterization of AM was reported for the first time by Kitamura et al. in 1993 from human pheochromocytomas of adrenomedullary origin, identifying it as a potent vasodilator (8). The *AM* gene encodes for a 185-residue preprohormone composed of two bioactive peptides: AM and proadrenomedullin N-terminal 20-residue peptide (PAMP) (9, 10). The preprohormone is firstly processed into proadrenomedullin, from which 20 amino acids at the N-terminus later form PAMP. The remaining AM precursor is successively converted into a C-terminal glycine extended intermediate of 53 amino acids (AM-gly) with scant activity –≈5% of AM effect–, and further into the 52-amino acid biologically-active form by α-amidation of the tyrosine at the carboxy terminal by a peptidylglycine α-amidating monooxygenase (PAM; EC, 1.14.17.3 **Figure 1A**) (11). Together with the amidated C-terminus, an intramolecular disulfide bond in the N-terminus giving rise to a ring of six or seven residues represent the distinctive structural feature of the calcitonin-like peptide family, which, besides AM, includes calcitonin, the calcitonin gene-related peptide (CGRP), amylin (AMY), and intermedin (or AM2). It is interesting to note their limited sequence homology as evidenced in **Figure 1B**. Also, despite structural likeness and the fact they share some biological activities, their main physiological roles are diverse (12).

Calcitonin is a 32-amino acid peptide encoded by the *CALCA* gene secreted by parafollicular cells –also known as C cells– in the thyroid gland. It plays a key function in the physiological homeostasis of serum Ca^2+^ inducing its absorption by bone tissue, and hence, hypocalcemia. CGRP results as an alternative splicing of the *CALCA* gene mRNA transcript (αCGRP) in the nervous system (13, 14), or as the direct (only mature) product of the *CALCB* gene (βCGRP) (15). CGRP –both isoforms– is widely distributed along the gastrointestinal tract and the central and peripheral nervous systems (16, 17). It is synthesized and released by sensory nerves and has much prominence in afferent neurotransmission. It also induces potent and long-lasting dilation of microvasculature of the nervous system and is involved in nociceptive signaling in migraines (17, 18). It likewise induces hypocalcemia with moderate potency (19). AMY, also referred to as islet amyloid polypeptide (IAPP), possesses a significant level of homology with CGRP, sharing 16 of 37 amino acids (20). AMY was originally identified in the pancreas, where it is co-synthesized, co-packaged, and co-released with insulin from islet β cells (21, 22). Nevertheless, further studies also localized this hormone in areas of the central nervous systems implicated in metabolic control, such as the hypothalamus (23). AMY deposits in type II diabetes are associated with the illness progression (24). In all, AMY is involved in the regulation of energy metabolism by mainly activating and modulating the satiating effect and stomach emptying through the central nervous systems (23, 25, 26), and antagonizing anabolic activity of insulin (4, 27). It also has a potent hypocalcemic activity (28).

AM displays several biological effects, in part due to its almost ubiquitous tissue expression. Apart from malignant and normal adrenal medullary cells, Kitamura et al. detected significant immunoreactive amounts of AM in the lung and kidney (8). Subsequent investigations reported AM expression in neurons and glial cells, but also in blood vessels (endothelial and smooth muscle cells), cardiomyocytes, macrophages, retinal epithelium adipose tissue, and different cancer cells. In fact, it is thought that all body tissues are able to secrete this peptide (29–31). In all cases, AM is not accumulated in vesicles, but its gene expression, synthesis, and subsequent secretion occur immediately in response to a vast series of biochemical (e.g., hormones and LPS) and physical (e.g., hypoxia and shear stress) cell type-dependent stimuli. In the context of this wide distribution of AM-secreting tissues, adrenal production is relative. Indeed, although present in lower concentrations (<1.5 fmol/mg), the total amount of AM in lung and kidney is higher than that produced by the adrenal medulla (≈150 fmol/mg) (8). Plasma concentration of total AM in humans (the sum of the less active, AM-gly and the mature active C-term amidated AM) varies from 2 to 20 fmol/ml (8, 32–35), being significantly increased in patients suffering from hypertension, chronic renal failure, heart failure, obesity, arteriosclerosis, and/or sepsis (30–37). However, it is worth noting that AM-gly is the main endogenous form of immunoreactive blood-circulating AM (38). In fact, the elevated concentration of total AM observed in hypertensive patients would be caused by AM-gly since plasmatic mature AM shows comparable levels between hypertensive and normotensive subjects (38). Kitamura et al. propound three non-mutually exclusive hypotheses to explain such paradoxical findings. 1) Most of the mature AM would act *in situ* by binding cell membrane receptors and thus a little fraction would be released to blood. 2) Alternatively, the scarce activity of AM-gly would not only facilitate its diffusion into peripheral vessels, but also extend its half-life. 3) Since the mature AM is thought to derive from AM-gly, both the location and activity of the PAM would therefore have a key role in regulating the AM/AM-gly ratio (38). In this sense, authors suggest that the misalignment in terms of PAM and AM expression amongst different tissues could explain the relatively high plasmatic levels of AM-gly. However, their hypothesis is based on the distribution of α-amidilating enzymes in rat (39), which could differ in humans as observed in the case of the lung (40).

AM acts as a circulating hormone but it also elicits multiple biological activities in a paracrine and/or autocrine manner. Its effects are of importance for cardiovascular homeostasis, growth and development of cardiovascular tissues, modulation of the lymphatic flow, regulation of body fluids and diabetes mellitus (8–10, 41–48). Systemic AM administration has been demonstrated to reduce arterial pressure, decrease peripheral vascular resistance, and increase heart rate and cardiac output (8, 49). Moreover, AM and PAMP act as potent angiogenic agents, being necessary for the maintenance of functional membrane microvasculature integrity (45, 47), and regulate lymphatic edema drainage promoting a faster healing of epithelial wounds (50, 51).

In the context of body fluid volume and renal function, AM exerts a tight control of the hypothalamic-pituitary-adrenal axis at all levels (33, 52). AM and its receptors are abundantly expressed in the central nervous system and its cellular components (53). It plays an important role in the regulation of specific blood-brain barrier properties (54), it also increases preganglionic sympathetic discharges (55) and exerts several neuroprotective actions against ischemic damage (56). Furthermore, relatively recent studies suggest that AM may be involved in the neuroendocrine response to stress and nociception (57).

In the digestive system, AM immunoreactivity is widely distributed in the mucosal and glandular epithelia of the stomach, esophagus, intestine, gallbladder, bile duct, and acini of the pancreas and salivary glands (58). It also regulates insulin secretion, directly acting on pancreatic cells (10), and is a potent inhibitor of basal gastrin-stimulated HCl secretion (59). Moreover, it has emerged as a novel and promising therapy for digestive pathologies related with inflammation such as gastric ulcers (60) and inflammatory bowel diseases (61). This is related to the local and systemic anti-inflammatory actions that AM is able to exert (62, 63). For example, it has been demonstrated that AM inhibits the secretion of pro-inflammatory cytokines into the medium by peripheral blood monocytes (64) and plays a role in the evolution of Th1/Th2 cytokine balance, decreasing pro-inflammatory cytokine levels (IL-6, IL-10, TNF-α, IFN-γ) (65–67). In addition to the regulatory role on immune cells, AM also decreases endothelial permeability, thus reducing the formation of inflammatory exudates (64). Likewise, it has been found in all epithelial surfaces that separate the external and internal environment and in all body secretions (68). This wide distribution suggests the possibility that AM has an immunity-related function. In this sense, it has been proven that AM displays potent antimicrobial action against Gram-positive and Gram-negative bacteria (69).

Despite other members of this family, AM possesses limited effect in bone tissue and therefore in calcemic regulation (12).

In 2004, two different groups independently reported the identification of an AM-high-homology peptide in humans. Takei et al., who had previously isolated this AM analog from pufferfish (70), called it AM2 (71); while Roh et al. employed the term intermedin (72). AM2/intermedin is a 53-amino acid peptide resulting from the cleavage of a (148-amino acid) pre-prohormone (72, 73). Rho et al. also reported other two further alternative cuts able to generate 40- or 47-amino acid versions of the hormone (72). In mice, it is highly expressed in submaxillary gland, stomach, pancreas, intestines, kidney, lung, mesentery, thymus, spleen, ovary (but not in testis), and the immune system (71). Both Takei et al. and Rho et al. emphasize the relatively significant levels of the AM2/intermedin in the pituitary gland, which would regulate hormone secretion in an autocrine and/or paracrine manner (71, 72). Like AM, AM2/intermedin possesses a strong hypotensive effect in normal and in spontaneous hypertensive rats (72) as well as cardioprotective effects against myocardial ischemia/reperfusion injury in rats (73).

### Particularities of the AM (and Calcitonin-Like Peptide) Receptors

At this point, it is clear that AM and the other hormones of this family exert a broad variety of physiological effects by acting on diverse tissues and systems. What is striking is the fact that all these processes arise from their interaction with only two closely related proteins: calcitonin and calcitonin-like receptors (CTR and CLR, respectively). These are G protein-coupled receptors (GPCR) belonging to the B1 subfamily, which are linked with G_s_ and thereby signal through adenylate cyclase/cAMP (74). In principle, such apparent promiscuity could partially explain the superposition of biological activities displayed by some of these peptides. Nevertheless, the ligand affinity and thus pharmacological behavior of these GPCRs results from their additional heterodimerization with one of the three accessory receptor activity-modifying proteins (RAMP1, RAMP2 or RAMP3).

The three RAMPs share about 30% of homology in their ≈160-amino acid sequence that gives place to a common structure including a large extracellular N-terminal domain, a single transmembrane domain, and a very short cytoplasmic C-terminal tail (C-tail) (75). RAMPs are well conserved amongst mammals. Indeed, mouse and human amino acid sequence identity for RAMP1, RAMP2, and RAMP3 are 70%, 68%, and 84%, respectively (76). In the endoplasmic reticulum, either, RAMP1, RAMP2, or RAMP3 can pair with CLR or CTR acting as chaperones that confer ligand specificity and binding affinity. Simultaneously, that assembly allows the transport of the receptor complex to the plasma membrane. It has been shown that only RAMP3 bears a PSD-95/Discs large/ZO-1 homology (PDZ) domain in the C-tail which allows its interaction with different factors responsible for further endocytic receptor trafficking and recycling (77). Although RAMPs are ubiquitous throughout the body, there are differences in their tissue distribution, and the abundance of each isoform depends on the tissue type (78–80).

The pharmacology of the CTR –mediator of calcitonin effects– switches differentially in presence of RAMP1, RAMP2 or RAMP3, giving rise to the AMY heterodimeric receptors AMY_1_, AMY_2_, and AMY_3_, respectively. Analogously, the CGRP receptor results from dimerization of CLR with RAMP1, while AM receptors 1 (AM_1_) and 2 (AM_2_) correspondingly emerge from the assembly of CLR with RAMP2 or RAMP3. In parallel, AM2/intermedin exerts it effects through the CGRP receptor, AM_1_ and AM_2_, presenting the highest potency when binding the latter (72, 81). **Figure 1C** illustrates the CTR/RAMP- and CLR/RAMP-composed receptors and their respective affinity for the different ligands.

The modulating effect of RAMPs on the ligand affinity of CTR and CLR could be due to an allosteric change in the conformation of the GPCR, leading to the exposure of different binding epitopes; or to a direct contribution together with the GPCR with epitopes that interact with the hormones (82–85). As mentioned above, RAMPs do not only give rise to different AM-, CGRP-, and AM2/intermedin-preferring GPCRs, but also are involved in their trafficking from Golgi to the cell membrane, posttranslational modifications like glycosylation, signaling as well as recycling (82, 86, 87). Furthermore, it is worth mentioning that, although best studied and characterized, the modulating action of RAMPs is not exclusive to CTR and CLR. In this sense, RAMPs also operate as molecular chaperones and allosteric modulators of several GPCRs and their signaling pathways, including glucagon, growth hormone releasing hormone (GHRH), parathyroid hormone and chemokine receptors among others (82, 88, 89).

In the case of the AM and the CGRP receptors, full functionality requires a third element: the receptor-complement protein (RCP) (90, 91). This cytosolic component, bound through ionic interactions to the cell membrane, couples CLR with the G_s_-mediated signaling transduction pathway (92, 93). Different reports point out that RCP is not necessary for AM and CGRP receptors to recognize their respective ligands; however, they are not able to activate adenylate cyclase in its absence (91–93).

In all, **Figure 1D** illustrates the complex structure of AM receptors formed by the three aforementioned proteins: CLR acting as the ligand-recognizing component whose affinity and membrane location depends on the RAMP chaperone member, and the RCP as the transduction-coupling constituent.

Nowadays, it is well accepted that AM, as well as the remaining calcitonin-like peptides, exert their physiological and pathological activities through the complex receptors accordingly mentioned above and shown in **Figure 1C**. Nevertheless, as Hay et al. deeply reviewed and clearly explained, there have been some misunderstandings regarding the apparent capacity of two other particular proteins to act as AM receptors: RDC1 and GPR182 (also known as L1-R/G10D and, mistakenly, AMDR) (94). In the middle 1990s, both proteins were proposed as mediators of AM pharmacological effects (95, 96). Although further studies have not confirmed this (86, 97), the suggestion that RDC1 and GPR182 may be AM receptors persist to this day. Currently, RDC1 and GPR182 are not accepted to be genuine AM or CGRP receptors (94). RDC1 is considered an atypical chemokine receptor, also known as C-X-C motif receptor 7 (CXCR7) (98), while GPR182 has remained as an orphan GPCR so far (99).

### Involvement of AM Receptors in Mediating Physiological and Pathological Processes

AM_2_ and AM_1_ present comparable affinity for AM (81), thereby making difficult the distinction of their individual involvement in the aforementioned AM-mediated physiological activities. In these circumstances, their tripartite structure and the fact that CLR and RAMPs seldom migrate to the cell surface separately make the RAMP2- and RAMP3-focussed studies a good approach to differentiate the roles of AM_1_ and AM_2_. Naturally, when drawing conclusions, it must be always considered that RAMPs do not interact exclusively with CLR (and CTR).

Several studies indicate that murine AM (*AM^-/-^*), CLR (*Calcrl^-/-^*) or RAMP2 (*Ramp2^-/-^*) knockout embryos die at mid-gestation owing to severe edema as a consequence of altered angiogenesis and lymphatic vasculature (42, 45, 48, 100–104). Although there were divergences in terms of whether hemorrhage is present or not amongst the different models, which ultimately might be attributed to the employment of different mouse strains, the fact that the three knockout types resulted in a non-viable common phenotype not only suggests the importance of AM for embryonic development, but also evidences the necessity of the canonical AM_1_-mediated signaling in endothelial cells (105). In fact, this was confirmed by Kechele et al. achieving the survival of a *Ramp2^-/-^* fetus by engineering mice with endothelial-specific expression of *Ramp2* under the control of the VE-cadherin promoter (104).

Nonetheless, it raised the question of whether AM_1_ requirement was circumscribed to uterine growth. Concerning this, Koyama et al. further made use of interesting murine models enabling the study of *AM^-/–^* and *Ramp2^-/–^*derived phenotypes in adulthood (47). Specifically, they developed endothelial cell-exclusive *Ramp2* and *AM* knockout mice (E-RAMP2^-/-^ and E-AM^-/-^, respectively) as well as (tamoxifen) drug-inducible E-RAMP2^-/-^ mice (DI-E-RAMP2^-/-^), for *Ramp2* deletion induction in adults. Contrary to conventional *Ramp2^-/-^* mice, most (≈95%) E-RAMP2^-/-^ and E-AM^-/-^ animals died during the peri-natal period, and not *in utero*. The surviving ones developed systemic and interstitial edema, vascular abnormalities and vasculitis throughout age. These effects were comparable in the DI-E-RAMP2^-/-^ model but less severe in E-AM^-/-^ mice, possibly given the fact that AM is produced by a wide range of tissues (47). Of note, *Ramp2^+/-^* and DI-E-RAMP2^-/-^ animals presented a normal lymphatic system (48). In particular, *RAMP2^+/-^* mice showed substantially reduced fertility and other endocrinological alterations, including basal and maternal hyperprolactinemia, enlarged pituitary gland, accelerated mammary gland development, and altered skeletal properties among others (45, 101, 106). Nevertheless, these phenotypes were not reproducible in the *Calcrl^+/-^* genotype, implying they are not directly linked to AM/AM_1_ but respond to CLR-independent physiological roles of RAMP2 in other systems (106).

Tam et al. carried out a series of experiments in RAMP2-overexpressing mice demonstrating a prominent physiological role of AM_1_ in mediating the vasodilatory effects of AM in vascular smooth muscle cells (107). Likewise, their analysis indicates that AM would bind the CGRP receptor at high nanomolar-range concentrations once AM_1_ has been blocked. Interestingly, Pawlak et al. more recently drew a fully opposing conclusion. In a study employing *Calrl^+/-^, Ramp2^+/-^*, *Ramp1^-/-^*, *Ramp3^-/^*,*^-^* and *Ramp1^-/-^*/*Ramp3^-/-^* double-knockout mice, they demonstrated that the AM hypotensive effect would be mainly mediated by its action on CGRP receptor and secondarily on AM_1_ (108). These discrepancies must be clarified in future investigations.

On the other hand, *Ramp3^-/-^* mice presented normal fertility and angiogenesis in both embryos and as adults with no obvious phenotypic defects (48, 101). Postsurgical lymphoedema drainage was significantly delayed in these animals, pointing out AM_2_ as principal AM mediator in the regulation of lymphatic functionality (48). In addition, Dackor et al. observed that this genotype failed in gaining body weight in adulthood (101); however, this was not further confirmed by other authors (48). Interestingly, Dackor et al. also reported that *Ramp3^-/-^*, *Ramp2^+/-^*, and wild type genotypes showed comparable blood pressure. Noteworthy, this was not due to any compensatory up-regulation of either *Ramp2* or *Ramp3* gene expression, supporting the lack of functional redundancy of AM_1_ and AM_2_ *in vivo* (45, 47, 109, 110). In contrast, Ichikawa-Shindo et al., showed that *Ramp2^+/-^* mice displayed a slight but significant increase in blood pressure with respect to wild type animals accompanied by elevated compensatory levels of AM (45). Pawlak et al. also found elevated blood pressure in *Ramp3^-/-^* mice with respect to wild type ones, particularly in males (108). In line with this, Barrick et al., have observed that, in the RenTgMK transgene mice model of angiotensin II-induced chronic hypertension, renal damage, cardiac hypertrophy and cardiac apoptosis were substantially exacerbated in males over females when animals presented the *Ramp3^-/-^* genotype (109). The mechanistic reason that would explain these findings is not clear yet, but other RAMP3-regulated processes also seem to be influenced by gender (111). In fact, both *AM* and *Ramp3* may be induced by estrogens, RAMP3 even being associated with the origin of menopausal obesity, although independently of AM-signaling (111, 112). On the other hand, Zhao et al. have not observed estrogen-induced AM secretion in human endometrial primary cells (113).

Regardless of the apparent sexual dimorphism, *Ramp3* expression is consistently up-regulated in rodent models of cardiac hypertrophy, hypertension and heart failure, leading to the hypothesis that increased AM signaling through AM_2_ may have a cardioprotective aim. In this sense, Cueille et al. also reported increased RAMP3 and (RAMP1) in atria and ventricles from rats in a non-ischemic model of chronic cardiac insufficiency due to pressure overload caused by aortic banding for six months (114). No variations were observed in CLR and AM at that time. In a further independent study, on the other hand, the left ventricle of rats with aortocaval shunt-induced cardiac hypertrophy presented up-regulated gene expressions of *AM*, *Calcrl*, *Ramp2*, and *Ramp3* compared with controls five weeks after the surgery (115). In a similar work, Oie et al. found that basal *Ramp2* mRNA in rat ventricular cardiomyocytes and non-cardiomyocytes cells is significantly higher than *Ramp3* mRNA transcript. However, this pattern was drastically inverted one week after a congestive heart failure induced by left coronary artery ligation (116).

LPS-induced septic shock in mice resulted in a strong decrease of *Calrl* and *Ramp2* mRNA expression in lungs, accompanied by a substantial rise in the levels of RAMP3 in pulmonary tissue and the principal organs in immune system. This is thought to be a body response to dampen the inflammatory process (36, 110).

All these findings collectively suggest that AM_1_ is essential for angiogenesis, vascular homeostasis and embryonic development while AM_2_ is meaningfully involved in lymphatic system physiology and would be induced, under certain physiological or pathological conditions, to adjust the CLR signaling. It is also quite plausible that the particular PDZ domain-bearing C-tail of RAMP3, enabling its interaction with molecules involved in trafficking of GPCRs, could be of crucial importance in the precise and dynamic regulation of AM_2_ availability in the cell membrane.

The signal transduction pathways activated by AM in order to regulate processes such as vasodilation, cell survival, proliferation, migration, and vascular cord-like structure formation vary between species, organs, tissues, and cells. However, the main signaling pathways whereby AM exerts its actions involve cAMP, Akt, mitogen activated protein kinase (MAPK)-extracellular signal regulated protein kinase (ERK), and the tyrosine phosphorylation of focal adhesion kinase (P125^FAK^) (117, 118).

## AM action in Malignant Context

### Roles of AM in Cancer

Since its discovery from human pheochromocytoma extracts, cumulative clinical evidence has revealed the association between AM and a variety of tumor types, showing that it is expressed by malignant cells, endothelial cells, pro-angiogenic cancer-associated fibroblasts (CAFs) and tumor-associated macrophages (TAMs), immature monocytic cells including TIE2+ monocytes, VEGFR1+ hemangiocytes, and CD11b+ myeloid cells within the tumor microenvironment (119–122).

Elevated circulating AM has been reported in the plasma of patients with lung and gastrointestinal cancers (123), as well as in untreated Cushing’s disease due to pituitary ACTH-producing adenoma, as compared with normal subjects (124). The increase in the former case seems related to ACTH production and immediately normalizes after surgical excision of the tumor. AM overexpression could be considered as a compensatory response to the elevated circulating cortisol. However, it is worth remarking that it was observed that AM concentration was about double on the side where the adenoma was localized, suggesting it may be directly produced by the tumor (124).

Patients with intraocular or orbital tumors presented significantly elevated AM mRNA levels in the malignant tissues in comparison with those having proliferative vitreoretinopathy, proliferative diabetic retinopathy, preretinal macular fibrosis, and acute retinal necrosis; indicating that AM may play a specific role in the pathogenesis of these cancers (125).

AM expression has been also described in human malignant pleural mesothelioma (MPM) and melanoma biopsies (119, 126). In colorectal cancer, the AM mRNA transcript level has been suggested as a useful marker for predicting high risk for relapse and cancer-related death in patients who undergo curative resection (127). Moreover, it has been found that plasma AM levels positively correlated with malignancy (120). Notably, increased expression levels of AM have been observed in samples harboring a mutation in *KRAS*. Instead, with the exception of scattered positive staining at the base of the glands in neuroendocrine cells, normal colon tissues were negative for AM expression (121, 122).

High expression of AM was found in human glioma samples, especially in the most aggressive form, namely, glioblastoma, whereas it was low in anaplastic astrocytoma and barely detectable in the low-grade astrocytoma and oligodendroglioma (128, 129).

Increased in *AM* mRNA expression was also reported in samples from those prostate cancer patients presenting the worse prognoses, as indicated by high Gleason’s scores, while practically absent in tissues samples collected from benign pathologies (130). Similarly, different works indicate that elevated AM expression associates with higher incidence of metastasis, larger residual size of tumors after cytoreduction, and shorter disease-free and overall survival time in epithelial ovarian cancer patients (5, 131), and correlates with tumor grading and metastasis in osteosarcoma (4) and hepatocellular carcinoma (23, 132).

Analogously, elevated tissue *AM* mRNA is a distinguishing feature of clear-cell renal carcinomas compared with other kidney tumors, and it is associated with an increased risk of relapse after curative nephrectomy due to this type of carcinoma (133, 134). Actually, Michelsen et al. reported significantly increased plasma AM concentrations in patients with renal malignant disease compared with healthy controls (133). Nevertheless, as mentioned above, renal failure is associated with elevated blood levels of AM. Therefore, impaired function of the affected kidney may be partially responsible for AM up-regulation. For this reason, plasma AM may not be suited as a tumor marker for renal cancer (80, 134).

Quantification of plasmatic AM concentrations showed no substantial difference between breast cancer patients and healthy women (135). Nonetheless, a significant positive correlation between tumor diameter and plasma AM levels was observed, suggesting not only that the breast malignancies were actually the source of the circulating AM, but also that tumors require a critical size until a noteworthy increase of secreted AM is detectable in blood (120, 135).

The *AM* gene has been found markedly overexpressed in patients with pancreatic cancer when compared with controls with benign/cystic pancreatic diseases or pancreatitis (136, 137). Likewise, the expression of *AM* was higher in subjects with pancreatic cancer and diabetes mellitus as compared with those not suffering from the former. These increased levels were detected in malignant tissue, at both RNA and protein levels, and as circulating AM (137).

Preclinical research does not only support these findings, but also has made great efforts in order to explain them. In this regard, it is worth remarking upon the work carried out by Prof. Ouafik’s group showing a correlation between AM expression and disease stage, progression or vascular density in the context of human glioblastoma (128), prostate (130, 138, 139), colon (121), MPM (126), pheochromocytomas (140), lung (141), and renal (134) cancers. In summary, these as well as studies from other laboratories indicate that AM itself does not cause cancer but can contribute to its pathogenesis in three main ways: 1) directly stimulating cell growth and inhibiting apoptosis (142–144); 2) inducing tumor angiogenesis and lymphangiogenesis, thereby supplying nutrients and oxygen to the tumor while boosting metastasis (45, 103, 126, 139, 145); 3) and/or changing the phenotype of cells, leading them to exhibit a more aggressive behavior (126, 138).

With respect to the first mechanism, several studies have demonstrated that AM exposure enhances cell growth and/or invasion in *in vitro* and/or *in vivo* models of breast (146), colon (121), prostate (139), renal (134), and glioblastoma (128) tumors, amongst others. The intrinsic processes behind these effects are not completely clear yet. Nevertheless, AM has proven to be able to inhibit apoptosis of tumor and endothelial cells by down-regulating pro-apoptotic factors such as fragmented PARP, Bax, and activated caspases (143, 144, 147).

In addition, AM protects malignant cells from hypoxia-induced cell death by up-regulation of Bcl-2 in an autocrine/paracrine manner in endometrial carcinoma and osteosarcoma cells (144, 148). In the former, the mechanism involves activation of the MEK/ERK1/2 signaling pathway (148). Similarly, AM was able to significantly increase proliferation and invasiveness of androgen-independent prostate cancer cell lines through stimulation of cAMP and the activation of the CRAF/MEK/ERK/MAPK pathway (139).

Furthermore, intraperitoneal injections of AM stimulated the growth of prostate androgen-dependent cell-derived tumors in castrated animals, suggesting that the peptide might be involved in tumor resurgence after testosterone ablation as in the case of patients treated with androgen-deprivation therapy (138).

Greillier et al. also reported increased activation of the CRAF/MEK/ERK/MAPK pathway in the AM-induced proliferation and invasiveness of the MPM-derived MSTO-211H and H2452 cell lines (126). In the case of hepatocellular carcinoma, the autocrine proliferative effect of AM in hypoxic conditions would be mediated by the PI3K signaling pathway (132). Likewise, the AM-activated PI3K/Akt pathway has been proved to protect endothelial cells from the apoptosis induced by hyperosmotic mannitol therapy for brain edema (149).

More interestingly, AM could also indirectly modulate MYC activity by regulating MYC-associated factor X (Max). This is based on the results of Shichiri et al. in quiescent rat endothelial cells, in which AM stimulated the expression of Max without affecting MYC, and so prevented apoptosis triggered by serum deprivation (150). AM-induced MYC/Max perturbation may result in favor of anti-apoptotic and proliferative effects of MYC.

On the other hand, tamoxifen is an anti-estrogen employed in the treatment of hormone-dependent breast cancer. A major concern of this therapy is its proliferative-inducing effect in endometrium, which significantly increases the risk of malignization with the long-term intake (151). The mitogenic and anti-apoptotic properties of AM point to this peptide as the principal intermediary of tamoxifen’s adverse endometrial effects through non-canonical estrogen receptor-mediated mechanisms (113, 144, 152). Partial agonistic effects of tamoxifen on the estrogen receptor would be able to stimulate AM transcription *via* an activator protein-1 (AP-1)-directed pathway rather than estrogen signaling response elements in a cell type specific manner (113).

AM activity also appears to extend beyond the malignant cells, being able to act in the tumor environment. There, it may reduce the effectiveness of the immune system to destroy cancer cells by decreasing the expression of pro-inflammatory cytokines and inhibiting the activation of the alternative complement pathway by binding to complement factor H (153, 154).

Infiltrations of myelomonocytic cells are common in the stroma of different tumors, such as pancreatic ductal adenocarcinoma (PDAC) and melanoma, and are related to poor prognosis (119, 120, 154). In their models of PDAC, Xu et al. demonstrated that AM promotes myelomonocytic cell migration and invasion and a pro-tumor phenotype through activation of the MAPK, PI3K/Akt, and NOS signaling pathways, as well as the expression and activity of the matrix metalloproteinase-2 (120). Comparable results were obtained by Chen et al. reporting not only that AM is involved in macrophage polarization –acquisition of a pro-tumor behavior–, but also that AM produced by TAMs is a central factor in macrophage-induced angiogenesis and melanoma growth (119).

More recently, CAFs have also been reported to play a key role in secreting and mediating AM pro-metastatic and angiogenic effects in melanoma (155), pancreatic (156), and breast (157) tumors.

The best-studied activity of AM in the tumor environment is the induction of neovascularization. In this regard, some works have shown that AM did not induce any direct mitogenic effect in the B16/F10 (melanoma), S180 (sarcoma), MDA-MB-231 (breast), A549 (lung), and pancreatic cancer cell lines; instead, when injected in mice, it would induce their growth as tumor masses by providing a suitable blood flow (119, 136, 141, 146, 158, 159).

Decreased O_2_ partial pressure and the resulting increase of HIF-1α have been implicated as one of the underlying pathways causing AM overexpression in human tumors (160). Hypoxia-stimulated AM expression has been observed in a broad variety of tumor cells, including endometrial (144), osteosarcoma (148), colorectal (121, 122), renal (161), hepatic (132), prostate and promyelocytic leukemia (162), pancreatic (136, 158), and so on. Actually, since tamoxifen has proven to induce hypoxia in xenografts, this has been proposed to be an alternative mechanism by which this anti-estrogen may induce AM in endometrium (144, 163). Thus, given the fact that localized hypoxia is an intrinsic hallmark of most solid tumors, it is not surprising that plasma AM is elevated in distinct common cancer types (133). It was described that AM expression correlates with both VEGF and HIF-1α in colorectal cancer (127). Furthermore, It was observed that AM could be an upstream modulator of the HIF-1α/VEGF pathway, suggesting that AM might induce angiogenesis through VEGF expression as consequence of JNK and AP-1 activation in epithelial ovarian cancer (6, 7). Analogously, several *in vitro* and *in vivo* studies have demonstrated the involvement of hypoxia, HIF-1α, AM, and AM receptors in HCC development, and how anti-AM therapeutic approaches would be of great value to deal with this disease (164–166).

On the other hand, it must be considered that the major sources of AM are not only malignant cells themselves, but also the vascular endothelium. This was clearly demonstrated in a work by Iimuri et al. in which S180 sarcoma tumorigenic cells presented more difficulty in developing tumors in AM-heterozygous knockout (AM+/-) mice –expressing ≈50% of circulating AM– compared to wild type ones (159).

AM-producing tumors are characterized by an increased vascular density (163) that correlates with the probability of metastasis occurring (167). Indeed, once trapped in the lymphatic capillaries, the angiogenic potential of AM-overexpressing cancer cells would facilitate neovascularization and hence macroscopic metastatic growth (146). In line with this, the analysis of AM expression and the clinicopathologic features of breast cancer patients showed that axillary lymph node metastasis markedly correlated with the AM protein levels in tumors (135). In colorectal cancer, *AM* was one of the most selectively upregulated genes in cells with a mutant *KRAS* under hypoxic conditions (122). Silencing of K-ras drastically reduced AM expression levels in hypoxia compared to control, indicating that AM is a key target for the K-ras oncogenic action in poorly oxygenated tumors. In addition, knockdown of AM suppressed tumor growth and impaired angiogenesis in colon tumor xenografts (122). Studies in HUVEC indicate that AM-induced pro-angiogenic effects would be mediated by PI3K/Akt, ERK, and P125^FAK^ (118).

Besides modifying the nature of TAMs –inducing a M2 phenotype–, AM expression has proven to be associated with more aggressive forms of glioma (128), prostate (130, 138), ovarian (5, 131) and breast cancers, generally correlated with increased metastatic capacity (5, 146). In prostate cancer, AM promotes the appearance of a neuroendocrine-like phenotype –usually presented in the clinic as an aggressive and drug-resistant form– in an androgen-regulated manner in the testosterone-dependent LNCaP cell line (138). These cells normally express both AM as well as AM_1_ and AM_2_. However, AM production is significantly increased upon the removal of androgen both in culture and *in vivo* (when injected in immune-suppressed mice), suggesting that paracrine/autocrine AM secretion represents a survival mechanism that prostate malignant cells employ to regulate neuron-like differentiation when facing androgen deprivation conditions. Such a phenotypic effect would be mediated by AM-induced nuclear translocation of cGMP-dependent protein kinase (PKG), and subsequent regulation of gene expression.

*In vitro* studies with colon cancer cells have shown the capacity of AM to promote a more invasive phenotype (122).

**Figure 2:**
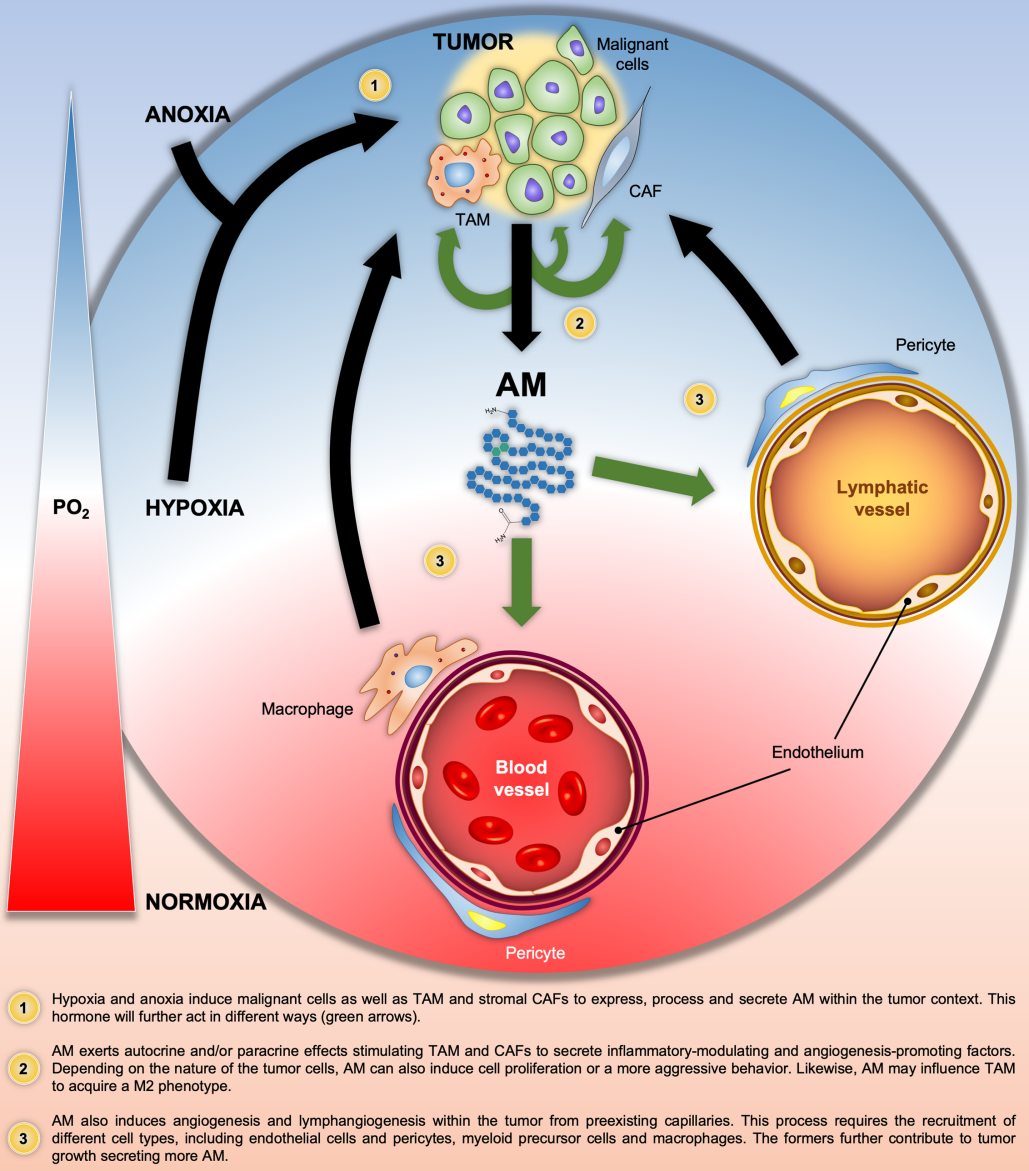
Graphical summary of the Adrenomedullin (AM)-related pathologic processes in solid tumors. Black and green arrows indicate the progression of the pathologic processes and the AM target cells, respectively. PO_2_, O_2_ partial pressure.

In summary, **Figure 2** illustrates all the above-mentioned AM autocrine/paracrine pro-tumor actions on malignant cells and/or different components of the tumor stroma.

### AM_1_ and AM_2_ in Cancer

Cancer cells employ circulating AM as well as that produced by themselves to promote tumor growth through the aforementioned mechanisms. In this section, we will review the role of key mediators of AM’s pro-tumor activity: AM_1_ and AM_2_. The findings discussed below are summarized in **Table 1**.

**Table 1:** Confirmed expression of AM, CLR, RAMP1, RAMP2, and RAMP3 in different tumors.

Cancer	Sample nature	AM	CLR	RAMP1	RAMP2	RAMP3	Report
**Adrenocortical carcinoma**	SW-13 cell line	+	+	NR	–	+	(168)
**Aldosteronoma**	Samples collected from patients	+	+	+	+	+	(142)
**Breast**	MDA-MB-231 cell line	+	+	NR	NR	+	(169)
	MCF-7 cell line	+	+	NR	+	+	(157, 170)
	CAFs collected from patient tumors	+	+	NR	+	+	(157)
**Colorectal**	Patient-derived tumors of clinical stages II, III, and IV	+	+	NR	+	+	(121, 122)
	HT-29	+	+	NR	+	+	(141)
	HCT116 cell line	NR	NR	NR	NR	+	(169)
**Fibrosarcoma**	HT1080 cell line	NR	NR	NR	NR	+	(169)
**Glioblastoma**	Patient-derived tumors	+	+	NR	+	+	(128)
	U87 cell line	+	+	NR	+	+	(128, 141)
	U373, U138, SW1783 and SW1088 cell lines	+	+	NR	+	SW1783	(128)
**Kidney**	Chromophobe and clear cell renal carcinomas	+	+	NR	+	+	(133, 134)
	786-O and BIZ cell lines	+	+	NR	–	+	(134)
**Liver**	Patient-derived hepatocellular carcinomas	+	NR	NR	+	+	(132)
	Huh-BAT and HepG2 cell lines	+	+	NR	+	+	
**Lung**	A549 cell line	NR	+	NR	+	+	(141, 146)
**Melanoma**	Patient samples	+	+	NR	+	+	(119)
	B16/F10 murine melanoma cell line	+	–	NR	–	–	
	B16/F10 xenograft TAMs	+	+	NR	+	+	(169)
	YU/PAC2 cell line	NR	NR	NR	NR	+	
	B16BL6 murine melanoma cell line	+	NR	NR	+	NR	(155)
**MPM**	Tumors collected from patients	+	+	NR	+	+	(126)
**Osteosarcoma**	F5M2 cell line	+	+	NR	+	+	(148)
**Pancreas**	Patient-derived PDAC	+	+	+	+	+	(136, 171)
	TAMs and PDAC-localized myelomonocytic cells	NR	+	NR	+	+	(120)
	Capan-1, Colo-357, T3M4, Mia-PaCa-2 and Panc-1 cell lines	+	+	+	+	T3M4	(136)
	BxPC-3 and PCI-35 cell lines	+	+	NR	+	NR	(158)
	PCI-10 and PCI-19 cell lines	+	–	NR	+	NR	
	PCI-43 cell line	+	–	NR	–	NR	
	PAN02 cell line	+	+	NR	+	+	(156)
	BxPC3, SU.86.86, Mia-PaCa-2, MPanc96, Aspc-1 and HPAC cell lines	+	–	+	+	–	(171)
	CFPAC-1 cell line	+	–	+	–	–	
	Panc-1 cell line	+	–	–	–	–	
	Mia-PaCa-2 cell line	+	–	–	+	–	
**Pheochromocytoma**	Clinical samples collected from benign and malignant tumors	+	+	+	+	+	(140, 142)
	Primary culture of tumor cells	+	+	+	+	+	(140)
**Prostate**	Prostate cancer of high-grade adenocarcinomas (Gleason's score > 7)	+	+	NR	+	+	(139)
	DU145 cell line	+	+	+	+	+	(130, 139, 162, 172)
	LNCaP cell line	+	+	NR	+	+	(138)
	PC3 cell line	+	+	+	+	–	(130, 146, 172)

The evaluation of the expression of AM CLR, RAMP1, RAMP2, and RAMP3 was determined at mRNA transcription level (PCR), and/or at protein level by Western blot and/or immunohistochemistry. NR, not reported.

Berenguer et al. observed that both AM_1_ and AM_2_ were responsible for mediating AM-induction of a neuroendocrine phenotype in the androgen-sensitive LNCaP cell line. Moreover, they described that, while the levels of CLR, RAMP2 and RAMP3 were not regulated by androgen status, levels of AM mRNA and immunoreactive AM increased 4- to 7-fold after androgen withdrawal *in vitro* and in LNCaP xenografts in animals after castration (138). Mazzocchi et al. did not find evidence of significant differences in the level of expression of either *Calcrl* or *Ramp1* and *Ramp2* mRNAs between prostate hyperplasia and cancer specimens. Nevertheless, they observed a clear higher expression of the *Ramp3* gene in the former group. Unfortunately, these authors did not specify the type and androgen dependency status of the cancers (172). Furthermore, these results were partially mirrored in *in vitro* studies with DU145 and PC3 androgen-independent prostate cancer cell lines. In this regard, *Calcrl*, *Ramp1* and *Ramp2* mRNA transcripts were detected in both cell types. In contrast, *Ramp3* expression was restricted to the DU145 cells, indicating that they resemble prostate cancer epithelial cells more closely than the PC3 cell line. Of note, AM showed proliferation-inducing and anti-apoptotic activities only in DU145 cells (130, 139, 172), pointing at AM_2_ as the mediator of such effects. This finding was supported by the fact that the AM antagonist CGRP8-37 was more effective than AM (22-52) inhibiting DU145 proliferation. It is well known the higher selectivity of CGRP8-37 for AM_2_ with respect to AM_1_ (172).

In clinical samples of renal cell carcinoma, RAMP3 has shown to be predominantly expressed in inflammatory cells associated with the tumor whereas RAMP2 was mainly localized in the malignant cells (134). On the other hand –and as part of the same study–, although authors suggest that the *in vitro* cell proliferation, invasion, and migration activated by exogenous AM would be mediated by both AM_1_ and AM_2_, RAMP2 was barely detected in the renal cancer cell lines employed (786-O and BIZ) (134), indicating that they may not be representative models of that cancer.

Chen et al. observed that *in vivo* tumor growth is significantly attenuated by AM antagonists in the murine B16/F10 melanoma cell line, even when components of the AM receptors were not detected (119). In contrast, TAMs were found to express not only AM, but also CRLR, RAMP2, and RAMP3. These cells are thought to be the main producers of hypoxia-induced AM within the tumor masses and so the mediators of angiogenesis. Moreover, the peptide will act in a paracrine and autocrine way inducing the acquisition of an M2 phenotype.

In the clinic, most human melanoma tissues were positive for CRLR, RAMP2, and RAMP3 –apart from AM–, while their expression levels were much lower in control healthy samples (119). Nevertheless, it would be of great value to determine the distribution of these proteins amongst different tumor cell types.

Ishikawa et al. obtained similar results in a series of five pancreatic cancer cell lines. In this respect, although all of them presented AM, only two (BxPC-3 and PCI-35) showed CLR and RAMP2 proteins. PCI-10 and PCI-19 cells showed RAMP2, and the PCI-43 cell line neither CLR nor RAMP2 (158). Interestingly, although anti-AM therapy had no effect in the *in vitro* proliferation of none the cell lines, it substantially abrogated PCI-43 cell-derived tumors *in vivo*, clearly implying that AM is acting on other components of the tumor environment.

A subsequent independent series of experiments performed by Keleg et al. in five other pancreatic cancer cell lines (Capan-1, Colo-357, T3M4, Mia-PaCa-2, and Panc-1) were partially in agreement with the previous results (136). The five assessed cell lines expressed AM, CLR, RAMP1, and RAMP2, whereas RAMP3 was detected in only one of them. Studies with patient samples showed increases in median *Calcrl* mRNA expression in PDAC in comparison to normal pancreatic tissues. In contrast, they observed >2- and >7-fold reduction in the median transcripts of *Ramp1* and *Ramp3*, respectively, in PDAC tissues compared to normal pancreas samples while no differences were detected in the expression of *Ramp2* between healthy and malignant tissues. Immunohistochemical analysis of CLR and RAMP1, RAMP2 and RAMP3 demonstrated moderate to strong staining in islets in the normal pancreas. In PDAC tissue, however, CLR colocalized with RAMP1 and RAMP2, and they were prominently expressed in malignant cells while RAMP3 was not detected. More recently, Xu et al. reported CLR, RAMP2, and RAMP3 in the pro-tumorigenic myelocytic cells associated with PDAC (120), which is in line with the capacity of AM to recruit different cell types reported by Kaafarani et al. (141).

Considering the AM pro-tumor effect, as well as RAMP1, RAMP2 and RAMP3 expression levels, it could be said that the results published by Ramachandran et al. in pancreatic cancer are partially in agreement with the previous ones. More specifically, none of the assayed cell lines (BxPC3, MiaPaCa-2, CFPAC-1, HPAC, MPanc96, Panc-1, Aspc-1, and SU.86.86) presented *Ramp3* or *CLR* expression. However, there are discrepancies in terms of *Ramp1* and *Ramp2* expression in BxPC-3, Panc-1, and Mia-PaCa-2 cells (171). Instead, authors attributed the AM-mediated pro-tumoral activity to the GPR182 receptor. They observed that by silencing GPR182 the basal growth was reduced as well as the AM-stimulated growth and invasive capacity of the malignant cells when compared with control shRNA (171). As in the case of the first report proposing GPR182 as an AM receptor, these findings have not been yet confirmed. Indeed, as concluded some paragraphs above, most of the cumulative evidence suggests that AM-induced tumor development in pancreatic cancer is mediated by AM_1_ and AM_2_. It is also noteworthy that they evaluated RCP expression, finding it in all the cell lines except CFPAC-1.

In all, these findings indicate that AM_1_ would be more relevant than AM_2_ in the pro-tumor action of AM in PDAC. Likewise, *in vitro* and *in vivo* evidence strongly suggest that AM induced tumor growth by mainly acting on tumor stroma components (120, 136, 158), proposing that the focus should not be on the malignant cells *per se* but in the peripheral stroma. In line with this, very recent experiments carried out by Dai et al. inoculating PAN02 tumor cells in the spleen of *Ramp3^-/-^* and DI-E-RAMP2^-/-^ mice not only demonstrated the active involvement of CAFs in the tumorigenic and metastatic capacity of these pancreatic cells, but also the different roles played by AM_1_ and AM_2_ (156). In this sense, when injected in DI-E-RAMP2^-/-^ animals, despite the tumor masses being significantly reduced and accompanied by defective angiogenesis, metastases to the liver were notably increased. In them, CAFs presented elevated RAMP3 and podoplanin (PDPN), a marker of lymphatic endothelial cells and lymphangiogenesis associated with a poor prognosis in various types of cancers. On the other hand, in *Ramp3^-/-^* mice, although tumor growth and angiogenesis were not affected (with respect to *Ramp3^+/+^* animals), tumors presented a less aggressive phenotype as indicated by the marked reduction in liver metastases as well as the number of PDPN-positive CAFs. In summary, in pancreatic cancer AM_1_ would be essential in promoting tumor growth and angiogenesis while antagonizing an AM_2_-mediated pro-metastatic effect (156).

This group had previously observed an increased propensity to lung metastasis in DI-E-RAMP2^-/-^ mice by generating B16BL6 melanoma cell line-derived tumors (155). Most tellingly, endothelial cell-specific RAMP2 overexpression in E-RAMP2 Tg mice strongly reduced lung metastasis and promoted survival. The authors hypothesized that the irregularly shaped, tortuous, and hyperpermeable vessels resulting from deficient angiogenesis (consequent of the DI-E-RAMP2^-/-^ phenotype) would be suitable substrate for the formation of pre-metastatic niches in distant organs (155). The sum of these findings suggests that a physiologically active AM/AM_1_ signaling system may be crucial to reduce the likelihood of metastasis in at least pancreatic and melanoma tumors.

Benyahia et al. more recently also demonstrated that CAFs extracted from invasive human breast adenocarcinomas are more competent than normal fibroblasts in enhancing MCF-7 cell growth by induction of stable vascularization when injected into immunocompromised mice (157). The authors demonstrated that AM is one of the CAF-derived factors responsible for endothelial cell-like and pericyte recruitment.

CLR, RAMP2 and RAMP3 have been also detected in glioblastoma tumors –both patient-collected samples and in immortalized malignant cell lines (128)– and in MPM (126). Indeed, expression levels in the former have shown to be much higher than in normal adjacent (pleural) tissue (126). Analogously, CLR, RAMP2, and RAMP3 immunostaining was scarcely detectable in the colonic epithelia of the crypts in normal tissue whereas the epithelial compartment in the well-differentiated adenocarcinoma samples showed strong staining for all these proteins (121). Evidence suggests that AM would act in a paracrine and autocrine manner in colorectal tumors; however, it is not yet clear the individual roles of AM_1_ and AM_2_ in the physio-pathology of the disease (121, 141). This is also applicable in hepatocellular carcinoma cell lines (132).

*In vitro* studies determined that the human SW-13 adrenocortical carcinoma-derived cell line only expressed *Calcrl* and *Ramp3*, while normal adrenocortical cells presented *Ramp1*, *Ramp2* and *Ramp3* mRNA transcripts, suggesting that the AM proliferative effects in the malignant cells are mediated by AM_2_ stimulation (168).

Clinical aldosteronoma samples were shown to express CLR, RAMP1, RAMP2, and RAMP3 mRNA, the levels of the two former being much higher than those of RAMP1 (142).

Studies with clinical samples and in primary culture of tumor cells have shown that, although benign pheochromocytomas secrete more AM than malignant ones, both share comparable expression levels of AM and (and CGRP) receptor components (CLR and RAMPs), RAMP1 being markedly abundant (up to 12-fold) with respect to RAMP2 and RAMP3 (140). Nevertheless, Thouënnon et al. suggest that RDC1 could be the predominant receptor for the autocrine effect of AM in this type of tumor basing their hypothesis on three pillars: 1) the elevated rate of expression of RDC1 over AM receptors components; 2) the fact that, among all genes examined in benign and malignant pheochromocytomas, *RDC1* was the only one that exhibited a significant differential expression between the two tumor subtypes, suggesting that it could be involved in malignant transformation; and 3) the assumption that AM is not pharmacologically active on the CGRP receptor. Considering the aforementioned strongly increased levels of RAMP1 over RAMP2 and RAMP3, authors presume that the competition for CLR would leave scarce possibility for the assembly of AM_1_ and AM_2_. If this is combined with the third pillar, there would not be a place for AM action in these cells. However, at this point it may be of particular interest to reconsider the, already mentioned, articles of Tam et al. (107) and Pawlak et al. (108) describing AM cardiovascular effects mediated through the CLR/RAMP1 complex. Considering that Thouënnon et al. do not give any proof of RDC1-activated signaling specifically in response to AM, the CGRP receptor seems more plausible as the AM target in pheochromocytoma. This is indeed supported by results obtained in the *RDC1*-downmodulated rat pheochromocytoma-derived PC12 cell lines suggesting that, although RDC1 may be involved in cell survival in serum deprivation conditions, it does not appear to be implicated in the AM-induced proliferative effect (140).

In line with the idea of alternative receptors, Zudaire et al. have reported that, in the tumor context, AM is able to induce mast cells to secrete different inflammation and angiogenesis mediators such as histamine and β-hexosaminidase by way of a process that would not be mediated by AM classical receptors, but, instead, by its interaction with other cell membrane proteins through electric charges. This effect is of particular pharmacological interest given the fact that it would escape from the action of AM_1_ or AM_2_ antagonists (160).

In all, except in the work of Ramachandran et al., AM pro-tumor effects always correlate with the occurrence of the necessary subunits to assemble functional AM_1_ and/or AM_2_, whether directly in cancer cells or in diverse stroma cell components (e.g., TAMs, CAFs, endothelium, etc.) (173). Likewise, there appears to be a differential predominance by one type of receptor depending on the type of cancer, which is of much importance in the eventual development of therapeutic approaches. In this sense, specific anti-AM_2_ agents would be more efficient in prostate cancers than in PDACs.

### AM-Targeting Agents and Potential Employment in Oncology

**Figure 3:**
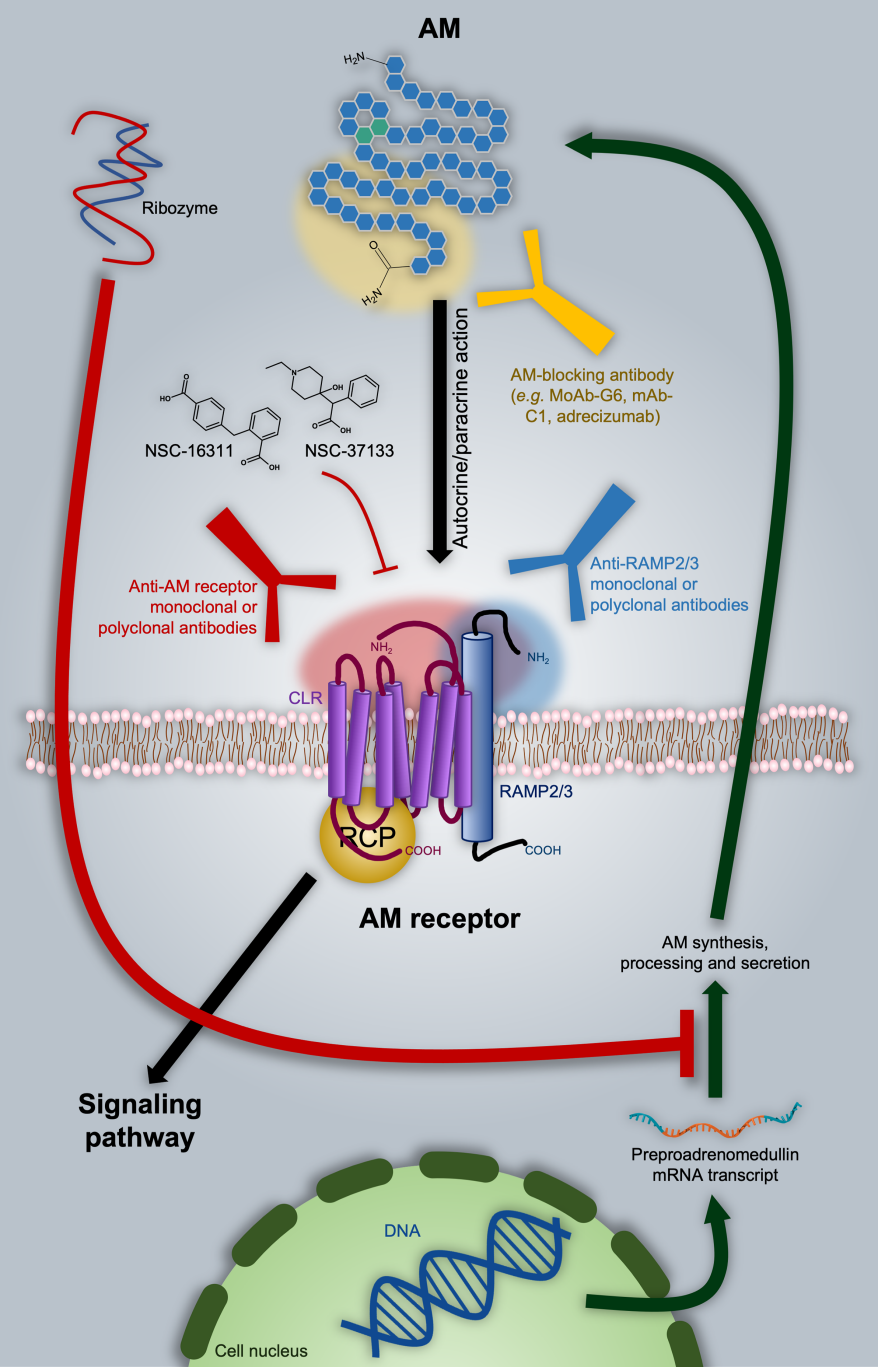
Adrenomedullin (AM)-based therapeutic approaches in oncology. As discussed in the text, one of the options is directly targeting AM with blocking antibodies, as in the case of the MoAb-G6, mAb-C1, and adrecizumab. A ribozyme has proven to be effective in diminishing AM expression. Nevertheless, it still presents significant disadvantages from the pharmacokinetic point of view. AM-receptor antagonists are represented by NSC-16311 and NSC-37133. These compounds have more favorable pharmacokinetic features with respect to peptides like AM (22–52)CONH_2_ (not shown). The binding of AM to its receptors can be also impeded by targeting the former with specific antibodies. In this respect, we introduce the possibility of employing anti-RAMP2 or anti-RAMP3 antibodies in order to get selective inhibition of AM_1_ or AM_2_ respectively. Potential epitope domains on AM and AM receptor components are indicated as shaded areas with the respective color of the targeting antibody.

Several strategies have been proposed to inhibit AM-mediated processes with potential application in oncology, including impediment of the AM-receptor interaction by antagonistic ligands, direct targeting of AM by specific blocking antibodies, and even regulation of the AM mRNA transcript. All these potential therapeutic approaches are discussed in the following paragraphs and summarized in **Figure 3**.

The first AM antagonists arose as result of structure-activity relationship (SAR) studies indicating that both the ring formed between amino acids 16 and 21 and the C-terminal amide are essential for the full demonstration of the AM hypotensive effect (174). On this basis, different laboratories undertook the search for truncated, pharmacologically inactive versions of AM able to antagonize it by competing for receptors. These investigations gave place to different types of derivatives: 1) AM (16-52)CONH_2_, AM (13-52)CONH_2_ (174), and AM (15-52)CONH_2_ (175) peptides with hypotensive and vasodilatory activity comparable to that shown by AM; 2) AM (22-52)CONH_2_ –also known as AM antagonist (AMA) and weakly selective for AM_1_ over AM_2_–, AM (1-52)COOH (176, 177), and AM (37-52)CONH_2_ (178), showing significantly lower receptor binding and pharmacological response; 3) AM (16-21), AM (16-31), AM (1-25) (179), and AM (11-26) (176), as inducers of increased systemic arterial pressure in rat; and 4) AM (33-52)CONH_2_ and AM (1-10)COOH, not able to bind AM receptors (174).

Together with the CGRP derivative CGPR (8-37), some molecules belonging to group 2 have been commonly employed as AM antagonists (172, 180), shown to reduce AM-mediated endothelial cell proliferation, migration, angiogenesis, tumor growth, and vascularization (5, 121, 157–159). However, whatever the pharmacological use the aforementioned peptides may have, their short half-life, low potency, and lack of selectivity circumscribe them only into the research field (175, 177, 180, 181).

It is worth mentioning that, at the moment at which most of those ligands were identified, the structures of the AM receptors were still unknown. Indeed, in many cases the evaluation of their antagonistic potency was determined by measuring their capacity to displace ^125^I-AM from the cell surface (174). Much effort has been made since then in order to get a molecular comprehension of the interaction of AM with its receptors for the further design and development of more selective and potent antagonists. In point of fact, nowadays it is accepted that while the C-terminal half of the AM binds the complex extracellular domain (ECD) formed by the N-terminals of CLR and RAMPs, the AM N-terminal portion –more precisely, residues 16-30– interacts with the CLR 7-transmembrane domain of the receptor, so stabilizing the conformation that will induce cytoplasmic signaling (83, 84, 182). In this context, Moad, and Pioszak identified the AM (37-52)CONH_2_ fragment as the minimal structure required to interact with the binding region of the ECD complex (178).

Robinson et al. carried out a series of studies with a number of peptide chimeras formed with AM, intermedin/AM2 and CGRP fragments, identifying several molecules with increased affinity compared to AM (22-52)CONH_2_ for AM receptors. Interestingly, the peptide called ‘C7’ [AM (22-36)-AM2 (23-30)-AM (37-52)] presented significantly higher selectivity for AM_2_ with respect to AM_1_ and the CGRP receptor, which is of great value from the pharmacological point of view in discriminating the individual role of each receptor in AM activity (183). In addition, these, and other authors, have demonstrated that selectivity is strongly established by the interaction of residue 74 in RAMPs with a series of a few amino acids in the C-terminal of AM (181, 183). More recently, Booe et al. identified that AM residues at position 45 and 50 are crucial in determining the affinity of AM for its receptors, as demonstrated by the significantly increased antagonistic capacity of AM (37-52)CONH_2_ after A45W and Q50W substitutions (84). Complementing this finding, Fischer et al. proved that the conformation of the ring 16-21 in AM is not only determined by the amino acid at position 22 (Thr), but also has a strong influence on the selectivity within the receptor system (182).

Unfortunately, so far, there are no further reports regarding the potential application in oncology of AM antagonists derived from these more recent studies. On the other hand, even if they resulted in more potent and selective molecules, the issue regarding their high metabolization rate would still not be solved.

The development of efficient, druggable small molecules is an alternative strategy to overcome the limiting poor selectivity and potency as well as short half-life often presented by peptide ligands. In this sense, Martínez et al. developed an elegant antibody-mediated method to screen for potential AM-antagonists in chemical libraries (180). Amongst the compounds identified, the piperidine derivative of phenylacetic acid, 16311 (also known as NSC-16311), elevated blood pressure when administered to normotensive (anesthetized) rats. On the other hand, the benzoic acid derivative, 37133 (also referred as NSC-37133), had no hemodynamic effects (180). In a later publication, the same group reported that both NSC-16311 and NSC-37133 as well as several other analogs exerted anti-proliferative effects in the human breast cancer cell line T47D, probably related to their AM-antagonistic behavior. In line with this, the AM-antagonistic action of NSC-16311 was further employed by Portal-Núñez et al. to demonstrate the AM contribution to the carcinogenic effect of tobacco combustion products in pulmonary tissue (184). More recently, Siclari et al. proved that NSC-16311 and NSC-37133 may be effective against breast cancer bone metastases due to their selective blockade of the osteolytic and pro-tumor-invasive actions of AM (146).

Taking advantage of the screening assay developed by Martínez et al. and complementing it by 3D quantitative SAR (3D-QSAR) and cell signaling studies, Roldós et al. further identified critical functional groups to be considered for the design of this type of ligands (185). Specifically, an aromatic ring, a hydrogen bond donor and a free carboxylic group seems to be essential to get AM-negative modulators to be eventually assessed as antiangiogenic and anti-cancer agents (185). However, it is notable that to date there are no clinical trials involving small-molecule AM antagonists.

In contrary, different selective small-molecule CGRP antagonists achieved clinical status for the treatment of migraine, including BIBN4096BS/olcegepant (186) (NCT02194777, NCT02194322, etc.), MK-0974/telcagepant (187) (NCT01294709, NCT00432237, etc.), MK-3207 (188) (NCT00712725, NCT00548353), and the very recently Food and Drug Administration (FDA)-approved MK-1602/ubrogepant (Ubrelvy^®^) (189) (NCT04179474, NCT02867709, etc.). This indicates the potential for developing selective antagonists for other CLR/RAMP heterodimers by exploiting key residue differences amongst RAMPs. In line with the aforementioned relevance of residue 74 in determining the selective interaction of RAMPs with their respective ligands, the tryptophan located in this position is crucial in mediating the binding of these compounds to RAMP1 (188). Instead, RAMP2 and RAMP3 share a glutamic acid residue at this position, indicating that such a class of anti-CGRP compounds may not be suitable scaffolds for the development of AM antagonists with oncological value. Even more so when taking into account that they were abandoned after some trial participants showed signs of hepatotoxicity (81)

On the other hand, the list of anti-CGRP agents with therapeutic action in migraine is not limited only to antagonists of the CLR/RAMP1 receptor, but also contains the commercially available, CGRP-neutralizing antibody fremanezumab (Ajovy^®^) (190) (NCT03308968, NCT04041284, etc.). Both mono- and polyclonal AM-blocking antibodies have been preclinically employed to inhibit AM pro-tumor and/or pro-angiogenic activity in glioblastoma (128), melanoma (119), lung (170), prostate (138, 139), ovarian, breast, and colon (121, 170) cancer models. Just to give examples, MoAb-G6 (170) and [anti-AM-(46–52)] mAb-C1 (191) are some of the monoclonal antibodies tested in these studies. Of note, no sign of toxicity was observed with this therapy (139). Nevertheless, the most relevant AM-blocking antibody is adrecizumab, currently under evaluation in cardiogenic and septic shock patients (NCT03083171, NCT04252937, etc.) (192). Results from these trials will be of great value given the fact that they will provide information on eventual side effects. Another question raised by the application of this approach concerns the impact competition with the AM-gly fraction –the most abundant circulating form of the hormone– may have on the bioavailability of the AM-neutralizing antibodies. To our knowledge, there are no reports in this respect.

Around 30% of all pharmaceutical products currently employed exert their therapeutic actions by modulating GPCRs (45, 75). The vast majority is represented by small molecules, then there is a minimal proportion composed by peptides, and, interestingly, the list is completed with only two FDA-approved monoclonal antibodies: mogamulizumab/Poteligeo^®^, employed in the treatment of mycosis fungoides or Sézary syndrome by targeting the CC chemokine receptor 4 (CCK4); and AMG 334/erenumab/Aimovig^®^, targeting CGRP receptor and prescribed for migraine prophylaxis (193–195). The approval of these two monoclonal antibodies gives promise to the potential of the currently available AM_1_-, AM_2_-, and CLR-targeting antibodies with proved tumor growth-inhibitory activity in preclinical models of colon, glioblastoma, PDAC, lung and breast cancer (120, 141, 157, 196). The anti-tumor effects of these antibodies are a direct consequence of their anti-angiogenic capacity. Indeed, treated tumor have been reported to appear pale or translucent, with diminished vasculature, permeability and recruitment of pericytes and macrophages and other myeloid cells (120, 141, 157, 196). Most tellingly, it must be noted that Kaafarani et al. did not observe signs of toxicity associated with long-term therapy (60 days). Histological analysis revealed no abnormalities of physiological vasculature in heart, liver, kidney, lung, and spleen. This could be partially explained by the specificity of the antibodies. In addition, it may mean that tumor angiogenic cells overexpressing AM_1_ and AM_2_ would become more susceptible to AM signaling blockade than the quiescent vasculature-associated cell components (141).

Over the years, several process-intrinsic challenges have limited the interest of the pharmaceutical industry in developing monoclonal antibodies against membrane proteins (195). However, the theory suggests that the complex structure of AM (and CGPR) receptors may compensate such restrictions, simultaneously offering encouraging advantages. A key factor in the development of such agents is to get antigens as close to the native conformation as possible, which is not a simple task for those proteins constantly moving immersed in a liquid film of phospholipids, as in the case of GPCRs. In this regard, it is thought that the mutual stabilization of the proteins (CLR and RAMPs) within the heterodimeric structure of AM_1_ and AM_2_ could allow them to remain longer in a physiologically relevant conformation. Moreover, the bipartite ECD creates a larger antigenic surface that increases the range of epitopes available to the antibodies, and so the chances of obtaining an appropriate therapeutic candidate (195). In contrast, the individual RAMP accessory proteins are more conventional single-pass receptors, and taken in isolation, represent more attractive targets, as recombinant expression of the extracellular domain is much less technically challenging. The RAMP components themselves represent individual therapeutic targets since they constitute strategic tools to define the pharmacological selectivity. In this sense, although its deficiency affects tumor vasculature and growth, RAMP2 does not appear to be a logical candidate for targeting given its role in preventing metastasis. In contrast, RAMP3 blockade in the context of a healthy AM/AM_1_ signaling is expected to considerably suppress tumor metastatic capacity (155, 156). Moreover, even when partial, RAMP2 deletion resulted in abnormalities in various vascular endothelial cells, which suggests attention must be paid regarding side effects when considering RAMP2 as the therapeutic target (155). On the other hand, *Ramp3^-/-^* mice did not show major phenotypic alterations (48, 110). Therefore, the incorporation of anti-RAMP3 agents in current therapeutic protocols in oncology may represent a promising anti-metastatic approach.

Lastly, a less conventional approach was proposed by Taylor and Samson, which consisted in the design of a catalytically active ribozyme that specifically recognizes and cleaves the preproadrenomedullin mRNA transcript (197). This molecule showed a good enough half-life to observe physiological variations both *in vitro*, decreasing AM content in cultured vascular smooth muscle cells, and *in vivo*, where reduction of AM resulted in exaggerated water drinking by rats after overnight water restriction or central administration of angiotensin II. Nevertheless, as an RNA-based molecule, stability, and route of administration are still drawbacks to consider in this strategy.

## Discussion

AM and VEGF share common features in that they are both hypoxically regulated, increase vascular permeability and are key pro-angiogenic factors. Indeed, their deficiency causes dysfunctional vascularization, even leading to embryonic lethality in mice when absolute (198, 199). It has been observed that both molecules can interact in an alternate complementary fashion in angiogenesis-involving processes. VE-cadherin is a protein component of endothelial cell-to-cell adherent junctions with a key role in the maintenance of vascular integrity, neovessel assembly and remodeling of pericyte-endothelial cell association. Dr. Ouafik and collaborators have demonstrated that AM blockade, using anti-AM or anti-AM receptor antibodies, increases endothelial cell permeability by inhibiting cell-cell contacts predominantly through disruption of the VE-cadherin/β-catenin/Akt signaling pathway (200). At a molecular level, AM blockade induces phosphorylation of VE-cadherin at the critical residue Tyr731, preventing binding of its cytoplasmic tail with β-catenin and thus disrupting vasculature structure. In contrast, the activation of the VEGF receptor 2 increases the phosphorylation of VE-cadherin VE at Tyr731 inducing loss of cell polarity and lumen formation, consequently allowing VEGF to stimulate endothelial cell proliferation (201). They also interact at the cell signaling level since VEGF activates a number of different intracellular signaling pathways, including ERK’s and PI3K/Akt to ensure endothelial cell survival and proliferation (202), which can be inhibited by AM blockade leading to endothelial cell death by apoptosis (personal communication of Prof. Ouafik’s group).

The general outcome of anti-angiogenesis therapy is still unsatisfactory, principally due to low efficacy and the development of tumor-acquired resistance and adverse effects. Although the causes are not clear, several hypotheses have been posed in order to explain the discrepancy observed in terms of the effectiveness of anti-VEGF treatments amongst (in-mice) preclinical studies and the clinic. One reason may be in the administration of higher doses in animals, that in general are younger and relatively healthier than patients, and for which therapy-related toxic reactions are often omitted (203).

Also, attention must be paid to the fact that anti-VEGF agents do not act directly on tumor cells, but on their supply of nutrients and O_2_. In this sense, residual hypoxia-resistant cells –usually associated with the cancer stem cell-like subpopulation– would act as a remnant source of VEGF and other factors that combined, would overcome the VEGF signaling blockade (203).

AM meets all the requirements to alternatively bypass VEGF inhibition, including a direct mitogen activity on malignant cells. Indeed, Gao et al. have demonstrated not only that *AM* expression was significantly (4-fold) increased in sunitinib-resistant renal carcinoma cells, but also that the treatment with AM (22-52)CONH_2_ markedly reduced the growth of tumors derived from those cells (204). In all, evidence points to AM as an alternative target in tumors resistant to anti-VEGF agents. In this respect, AM also offers a broader front of action within the milieu of solid tumors, being able to act as modulator of endothelial cell interactions with other endothelial cells and/or pericytes, recruiter of different cell types into hypoxic areas and stimulator of cancer cell proliferation. On the other hand, the mitogenic activity of VEGF is mainly restricted to endothelial cells.

However, such multitasking role carries increased risk of adverse effects. In this regard, hypertension and its associated secondary events (i.e., myocardial infarction and stroke) may be the main complication in anti-AM therapy, which is indeed an adverse effect observed in patients undergoing VEGF-inhibiting treatments. In general, however, anti-VEGF agents are well tolerated, especially when compared with chemotherapy; although, undesirable effects can limit their administration in some cases. These include venous and arterial thromboembolism, proteinuria, hemorrhage, gastrointestinal perforation and impaired wound healing (203, 205, 206). Actually, the former has been already reported in anti-AM therapy preclinical models. Mice-based studies indicate that the major adverse effects related to AM-inhibitory treatments may involve development of secondary lymphoedema (207, 208) and altered wound healing capacity (50). Both are of importance for those patients who undergo surgery and/or radiotherapy or might present a genetic susceptibility to the risk of developing secondary lymphoedema (208). However, they are manageable and usually pose a significantly lower risk than the tumors being treated.

One aspect to consider in any therapy is the capacity of identifying prognostic factors. Although in anti-VEGF regimens these types of molecules have remained elusive, VEGF itself has been postulated as general biomarker of angiogenic activity and tumor progression in cancer patients (129, 209). In this respect, increased circulating values of VEGF generally correlate with worse outcome (206, 209). In the case of anti-AM therapy, VEGF should be thought, at least, as the starting prognostic parameter to be evaluated.

We are of the opinion that there is mounting evidence of the potential of anti-AM strategies in oncology, representing more than just an anti-angiogenesis therapy, to merit further investigation. The efforts should be onwards focused on the discussion and development of efficient therapeutic approaches, taking also into account the feasibility of combination therapy with conventional chemotherapy. For example, Li et al. observed a significant potentiation of the tumor growth-inhibitory effect of cisplatin in AM-deficient liver cancer cells (210).

Considering the already described involvement of AM in different physiological activities, the extrapolation of a bevacizumab/Avastin^®^-like approach (directly targeting the ligand) may, in principle, not be a first option as a therapeutic strategy for AM. Another point to take into account in this respect is the fact that the AM-gly is the major circulating form of this protein. Thus, a potential antibody should be able to discern the AM mature variant in order to avoid the bioavailability-reducing competition with the inactive one, highlighting the AM amidated C-terminal as the antigenic epitope of choice.

As pointed out above, current clinical trials with adrecizumab will be more than interesting in addressing the feasibility of this strategy in oncology. Nevertheless, the blockade of AM receptors might represent a more suitable alternative. Moreover, the recent FDA approval of the CGRP receptor-targeting erenumab/Aimovig^®^ antibody encourages the application of the anti-AM receptors strategy in oncology. One of the key challenges regarding this approach is the ability to selectively target individual components.

Collectively, data discussed in the previous sections suggests that both AM_1_ and AM_2_ possess crucial roles within the tumor structure regulating diverse functional aspects of the cell components. In this context, RAMP2 and RAMP3 themselves represent target candidates. However, AM_2_ blockade could be presented as more advantageous by decreasing the chances of metastasis and having less impact on the physiological angiogenic processes. Furthermore, although it can be said it is a general feature of the AM system, the AM_2_ signaling pathway in particular appears to be in a physiological dormant state ready to assemble and operate in response to diverse pathological conditions. This could explain the low incidence of adverse effects observed when targeting this receptor in preclinical models.

In the light of the current evidence, AM inhibition is a promising first-in-class therapeutic strategy in oncology and its potential needs to be further explored for the development of selective pharmacological options in cancer patients.

## Author Contributions

RV: As primary author, conceived the original idea of the article, drafting and writing the paper, and made the figures. MR: primary author conceived the original idea of the article, and drafting and writing most of the paper. CB-D, AO’K, JG, OT, and KR: Participated in revisions to scientific content of specific sections of the manuscript. MB: Participated in the critical revision, editing, and writing of the manuscript. Provided revisions to scientific content of the manuscript. L’HO: Conceived the original idea and provided critical revision of the manuscript as well as the final approval of the version to publish. All authors contributed to the article and approved the submitted version.

## Conflict of Interest

Authors AO’K, JG, and OT are employed by the company Fusion Antibodies. RV, MR, and MB are stockholders of Early Drug Development Group.

The remaining authors declare that the research was conducted in the absence of any commercial or financial relationships that could be construed as a potential conflict of interest.

## Abbreviations

ACTH, Adrenocorticotropic hormone; AM, adrenomedullin; AM_1_, adrenomedullin receptor type 1; AM_2_, adrenomedullin receptor type 2; AM2, adrenomedullin 2 (intermedin); CAF, tumor-associated fibroblast. AMY, amylin; cAMP, cyclic-adenosine monophosphate; CGRP, calcitonin gene-related peptide; cGMP, cyclic guanosine monophosphate; CLR, calcitonin-receptor like receptor; CTR, calcitonin receptor; ECD, extracellular domain; ERK (1/2), extracellular signal regulated protein kinase (1/2); GPCR, G protein-coupled receptor; HIF-1α, hypoxia-inducible factor-1α; LPS, Lipopolysaccharides; MAPK, mitogen activated protein kinase; MPM, malignant pleural mesothelioma; PAM, peptidylglycine α-amidating monooxygenase; PAMP, Proadrenomedullin N-terminal 20 peptide; PDAC, pancreatic ductal adenocarcinoma; PDPN, podoplanin; PDZ, PSD-95/Discslarge/ZO-1; PKG, cGMP-dependent protein kinase or protein kinase G; RAMP, receptor-activity modifying protein; RCP, receptor-complement protein; SAR, structure-activity relationship; TAM, tumor-associated macrophage; VEGF, vascular endothelial growth factor.
